# Serpin genes *AtSRP2 *and *AtSRP3 *are required for normal growth sensitivity to a DNA alkylating agent in *Arabidopsis*

**DOI:** 10.1186/1471-2229-9-52

**Published:** 2009-05-11

**Authors:** Joon-Woo Ahn, Brian J Atwell, Thomas H Roberts

**Affiliations:** 1 Department of Chemistry and Biomolecular Sciences, Macquarie University, North Ryde NSW 2109, Australia; 2 Department of Biological Sciences, Macquarie University, North Ryde NSW 2109, Australia

## Abstract

**Background:**

The complex responses of plants to DNA damage are incompletely understood and the role of members of the serpin protein family has not been investigated. Serpins are functionally diverse but structurally conserved proteins found in all three domains of life. In animals, most serpins have regulatory functions through potent, irreversible inhibition of specific serine or cysteine proteinases via a unique suicide-substrate mechanism. Plant serpins are also potent proteinase inhibitors, but their physiological roles are largely unknown.

**Results:**

Six *Arabidopsis *genes encoding full-length serpins were differentially expressed in developing seedlings and mature tissues. Basal levels of *AtSRP2 *(At2g14540) and *AtSRP3 *(At1g64030) transcripts were highest in reproductive tissues. *AtSRP2 *was induced 5-fold and *AtSRP3 *100-fold after exposure of seedlings to low concentrations of methyl methanesulfonate (MMS), a model alkylating reagent that causes DNA damage. Homozygous T-DNA insertion mutants *atsrp2 *and *atsrp3 *exhibited no differential growth when mutant and wild-type plants were left untreated or exposed to γ-radiation or ultraviolet light. In contrast, *atsrp2 *and *atsrp3 *plants exhibited greater root length, leaf number and overall size than wild-type plants when exposed to MMS. Neither of the two serpins was required for meiosis. GFP-AtSRP2 was localized to the nucleus, whereas GFP-AtSRP3 was cytosolic, suggesting that they target different proteinases. Induction of cell cycle- and DNA damage-related genes *AtBRCA1*, *AtBARD1*, *AtRAD51*, *AtCYCB1;1 *and *AtCYCD1;1*, but not *AtATM*, was reduced relative to wild-type in *atsrp2 *and *atsrp3 *mutants exposed to MMS.

**Conclusion:**

Expression of specific serpin genes (*AtSRP2 *and *AtSRP3 *in *Arabidopsis*) is required for normal responses of plants following exposure to alkylating genotoxins such as MMS.

## Background

DNA damage results from exposure to specific chemicals in the environment, UV light, ionizing radiation and errors in DNA replication and proofreading. Plants utilize several pathways for DNA repair, including photoreactivation, nucleotide excision repair, base excision repair, mismatch repair and double-stranded break repair [1]. Methyl methanesulfonate (MMS) is a simple, direct alkylating agent recognized as a standard for genotoxicity assays of environmental pollutants [2]. MMS has been widely utilized as a γ-radiation mimic in the belief it causes double-stranded breaks (DSBs). A recent report found, however, that no MMS-mediated DSBs could be detected *in vivo *in yeast or mammalian cells, and those reported previously were almost certainly artefacts [3]. Molecular responses of organisms to alkylating phytotoxins are likely to be distinct from those to ionizing radiation.

Many intra- and extracellular processes in plant growth, development and responses to stress involve specific proteolytic enzyme activities. The *Arabidopsis *genome contains 656 known and putative peptidases [4] but the functions of only a tiny minority are known. Furthermore, little is known of the control of proteolytic activity *in planta *by endogenous peptidase inhibitors, including the serpins [5,6], which are one of seven families of peptidase inhibitors in *Arabidopsis *[4]. Serpins are metastable inhibitors with a unique, irreversible mechanism of action [7].

Almost all plant serpins studied are potent inhibitors of mammalian proteinases of the chymotrypsin family *in vitro *[8-12]. An *Arabidopsis *serpin, AtSerpin1 (At1g47710), was shown to inhibit the endogenous cysteine proteinase Metacaspase 9 (AtMC9) *in vitro *[11] but no other putative endogenous targets for plant serpins have been identified. Plant serpins are likely to function in direct defence against proteinases from pests and pathogens and in the regulation of endogenous proteolytic events, but no functions have been demonstrated [5,6].

Here we report the differential basal expression of six *Arabidopsis *serpin genes and the effect of MMS exposure of seedlings on the activity of *AtSRP2 *(At2g14540) and *AtSRP3 *(At1g64030), both specifically expressed in reproductive tissues. We determine the subcellular localizations of AtSRP2 and AtSRP3 and examine the growth responses of *atsrp2 *and *atsrp3 *mutants (vs wild-type) to MMS, γ-radiation and UV light treatments. Finally we compare the induction levels of cell cycle-related genes in the *atsrp2 *and *atsrp3 *plants compared to wild-type after exposure to MMS.

## Results

### *Arabidopsis *serpin genes are differentially expressed

**Figure 1:**
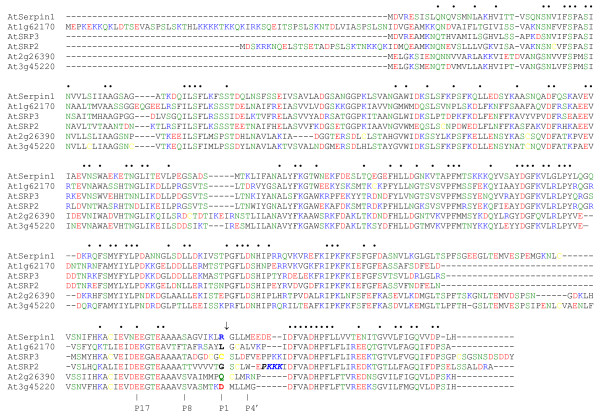
**Amino acid sequence alignment of full-length *Arabidopsis *serpins**. The alignment was created using ClustalW and edited. Locus numbers are given for some of the serpins. Amino acid residues are colour-coded: positively charged, blue; negatively charged, red; polar, green; cysteine, yellow; other residues, black. Dots above the alignment indicate residues identical in all six serpins. Putative positions of specific residues in the RCL are indicated below the alignment: P17 Glu, P8 Ser/Thr, P1 (shown in bold) and P4'. A gap, indicated by an arrow, between the P1 and P1' residues indicates the predicted site of proteinase cleavage. The predicted nuclear localization sequence for AtSRP3 is shown in bold italics. Large gaps in the sequences of the serpin at At1g62170, AtSRP2 and AtSRP3 (second block from bottom, right side) represent lack of a surface loop lying between helix I and strand 5 of β-sheet A (based on the human α_1_-antitrypsin structure); absence of this loop is not expected to affect folding to a metastable structure capable of inhibiting proteinases [5].

PSI-BLAST searching of the *Arabidopsis *genome revealed six predicted full-length serpins (~340–440 residues) [6]. The numbering system used for the RCL residues is that of Schechter and Berger (1967) whereby residues N-terminal to the proteinase cleavage site are numbered P1, P2, P3, etc and those C-terminal to the cleavage site are numbered P1', P2', P3', etc [13]. Reactive centre loop (RCL) sequences were aligned using the conserved P17 Glu, P14 Thr and P8 Ser/Thr, allowing the reactive centre P1 residue – the most important for inhibitory specificity – to be identified for each serpin (Figure 1). One of the *Arabidopsis *serpins (At1g62170) was predicted to be non-inhibitory (based on P10 Thr and P11 Val) but each of the five remaining serpins was predicted to be inhibitory [5] and has a unique reactive centre (Figure 1).

**Figure 2:**
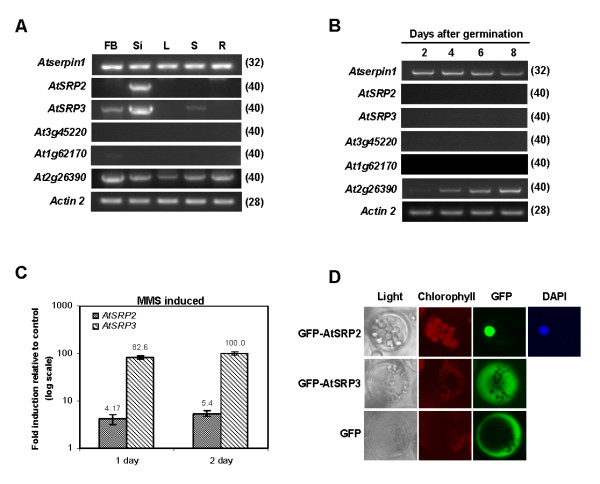
**Expression of *Arabidopsis *serpin genes**. (A, B) Semi-quantitative RT-PCR. Values in brackets indicate number of cycles. *Actin2 *served as a control. (A) Expression of serpin genes in mature tissues. RNA was extracted from 6-week-old plants. FB, flowers and flower buds; Si, siliques; L, leaves; S, stems; R, roots. (B) Expression of serpin genes in whole seedlings during early development (2–8 d). (C) Induction of *AtSRP2 *and *AtSRP3 *in response to MMS treatment of seedlings (5-d) after 1 and 2 d as determined by quantitative RT-PCR. Values are means +/- SE (n = 3). (D) Subcellular localization of AtSRP2 and AtSRP3. GFP fusion constructs with AtSRP2 and AtSRP3 full-length cDNAs, respectively, were transformed into *Arabidopsis *protoplasts. Bright-field, chlorophyll, GFP and DAPI-stained images are shown. Expression of GFP alone was a cytosolic marker. DAPI staining served as a marker for nuclear localization.

We examined basal transcript levels of the six serpins in mature tissues and in whole seedlings during development using semi-quantitative RT-PCR. *AtSerpin1 *(At1g47710) was the most highly expressed serpin in all tissues (Figure 2A). This gene and At2g26390 were ubiquitously expressed in the mature plants. In contrast, *AtSRP2 *and *AtSRP3 *transcripts were detected more specifically in siliques, with *AtSRP2 *also expressed very weakly in flowers and *AtSRP3 *in flowers and stems. At3g45220 and At1g62170 expression was not detectable in mature tissues. *AtSerpin1 *was ubiquitously expressed throughout seedling development (Figure 2B). Weak expression of At2g26390 was detected at day 2 and increased during days 4 to 8. At3g45220 and At1g62170 expression was not detectable in seedlings. *AtSRP2 *and *AtSRP3 *transcripts were detected at very low levels in seedlings (5 d) by quantitative RT-PCR (data not shown).

### *AtSRP2 *and *AtSRP3 *are upregulated following MMS treatment of seedlings

Wild-type (5-d) seedlings were transplanted to liquid media containing 200 ppm MMS and transcript levels of *AtSRP2 *and *AtSRP3 *determined by quantitative RT-PCR. *AtSRP2 *and *AtSRP3 *were induced ~5-fold and ~100-fold, respectively, after 2 d (Figure 2C). In a separate experiment, wild-type seedlings (5-d) were exposed to MMS for 1 and 3 h but no induction was detected. In untreated seedlings (5-d) and in seedlings 1 and 3 h after exposure to γ-radiation (125 Gy) no *AtSRP2 *and *AtSRP3 *transcripts were detected using semiquantitative RT-PCR (data not shown).

### Subcellular localization of *AtSRP2 *and *AtSRP3*

GFP-AtSRP2 was localized to the nucleus whereas GFP-AtSRP3 was localized to the cytosol (as was GFP alone, as expected) (Figure 2D). DAPI staining confirmed the identity of the nucleus.

### Identification of T-DNA insertion mutants

**Figure 3:**
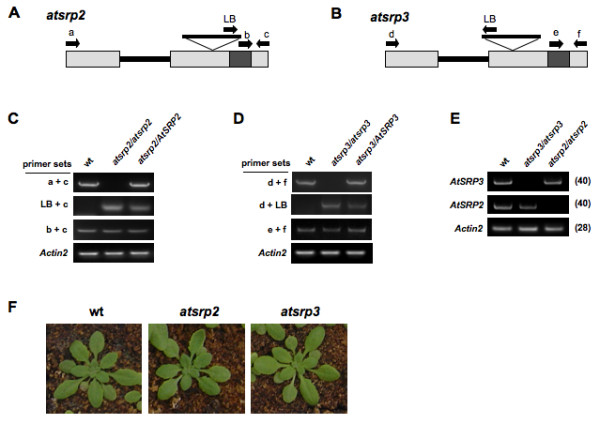
**Schematic representations of *atsrp2 *and *atsrp3 *T-DNA insertion mutants and identification of homozygous lines**. (A, B) Genomic sequences of *AtSRP3 *and *AtSRP2 *each contain two exons. Primers for screening of homozygous lines are indicated by letters a-f and LB (left border). Gray boxes indicate the RCL motif. (C, D) Genomic-PCR analysis for screening of homozygous lines using the primers shown in *A *and *B*. wt, wild-type. (E) Confirmation of knockout of *AtSRP2 *and *AtSRP3 *expression in each mutant via semi-quantitative RT-PCR. RNA was isolated from developing siliques. Values in brackets indicate number of cycles. *Actin2 *served as a control. (F) Phenotype of *atsrp2 *and *atsrp3 *mutants. Mutants and wild-type plants were grown in a growth chamber at 24°C. Photographs were taken at 4 weeks after sowing.

Two T-DNA insertion mutants (*SALK_088095 *and *SALK_072458*) corresponding to *AtSRP2 *and *AtSRP3*, respectively, were identified in the SIGnAl database. No other T-DNA insertion lines were available for these two genes. The *atsrp2 *and *atsrp3 *mutants have a T-DNA insertion on the right (Figure 3A) and in the middle (Figure 3B) of the second exon, respectively. Genomic PCR using primer sets corresponding to the 5' and 3' ends of each gene and to the left border of the T-DNA insert was performed to identify homozygous lines. The absence of full-length PCR product using the 5' and 3' primer sets indicated the presence of the T-DNA insertion (~4 kb) in both copies of the gene (Figure 3C and 3D). To confirm the knockout of each *AtSRP2 *and *AtSRP3 *transcript in the corresponding homozygous mutant, total RNA was prepared from developing siliques and RT-PCR performed. No transcripts for *AtSRP2 *and *AtSRP3 *were detected in the corresponding mutants, and there was no apparent compensation of *AtSRP2 *transcript levels in the *atsrp3 *mutant, or vice-versa (Figure 3E).

### *atsrp2 *and *atsrp3 *mutants grow faster than wild-type plants when exposed to MMS but not to γ-radiation or UV-C

**Figure 4:**
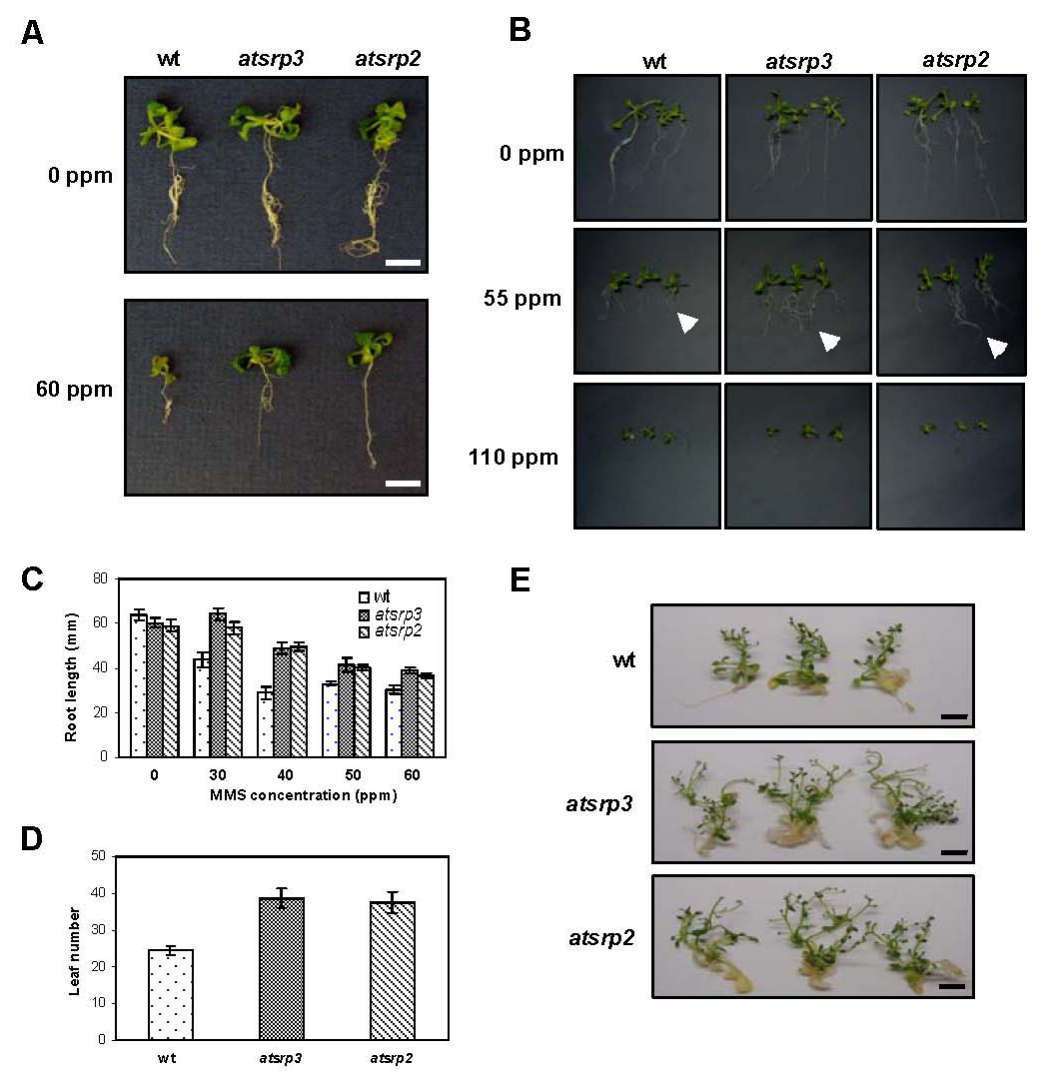
**Responses of *atsrp2 *and *atsrp3 *mutants to MMS**. B5 and half-strength MS (1/2 MS) media were used for seedling growth. (A) Vigour of *atsrp2 *and *atsrp3 *mutants compared to wild-type following treatment with MMS. Seedlings (5-d) of equal size were grown in 1/2 MS liquid media containing the indicated concentrations of MMS for 2 weeks. Size bars = 10 mm. (B-E) Seedlings (wild-type and mutants) were grown in the presence of the indicated concentrations of MMS on single plates to provide identical growth conditions. (B) Wild-type and mutant plants showing differential root lengths. Seedlings (5-d) were grown on B5 solid media. Arrows indicate root tips of mutants and wild-type plants (centre plant in each case) for comparison of root lengths. Photographs were taken on day 14. (C) Root lengths. Values are means +/- SE (n = 10). Seedlings (5-d) were grownon 1/2 MS solid media. (D, E) Seedlings (2-week) were transplanted to B5 solid media containing 55 ppm MMS. (D) Leaf number per plant. Counts were made at 4 weeks after transplantation. Values are means +/- SE (n = 4). (E) Wild-type and mutant plants showing different plant sizes. Photographs were taken 4 weeks after transplantation. Size bars = 10 mm.

**Figure 5:**
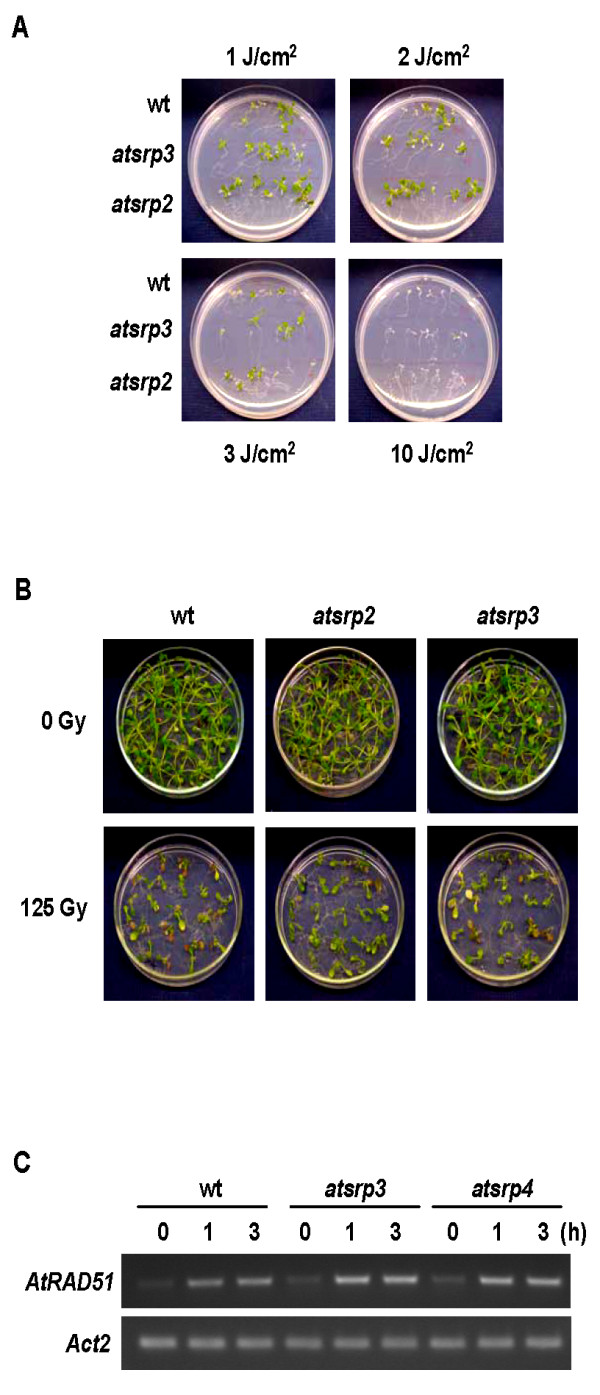
**Response of *atsrp3 *and *atsrp2 *mutants to UV radiation and γ-radiation**. (A) Seedlings (5-d) were exposed to UV radiation (1, 2, 3 and 10 J cm^-2^) and grown at 24°C for 3 weeks. Photographs were taken on day 21 after exposure. (B) Seedlings (5-d) were grown on B5 solid media and subjected to γ-radiation at doses of zero (control) and 125 Gy. Photographs were taken 2 weeks after irradiation. (C) Expression of DNA damage-related gene *RAD51 *in whole seedlings (5-d) following exposure to γ-radiation was determined using semi-quantitative RT-PCR.

Mutants *atsrp2 *and *atsrp3 *were phenotypically normal under standard growth conditions (Figure 3F). Both mutants produced apparently normal siliques, seeds (number and size), and wild-type germination rates (data not shown), indicating that *AtSRP2 *and *AtSRP3 *are not essential for meiosis under standard growth conditions. 1/2 MS and B5 media were used to examine responses of mutants to MMS versus those of wild-type (similar results with both media). Surprisingly, when *atsrp2 *and *atsrp3 *plants were exposed to MMS, both mutants grew consistently more vigorously than wild-type plants given the same treatment (Figure 4A). In the absence of MMS, there was no significant difference in root growth between wild-type and mutants grown on solid media. For plants grown on B5 media in the presence of ≥ 55 ppm MMS, however, roots of *atsrp2 *and *atsrp3 *mutants grew longer than wild-type roots over 2 weeks (Figure 4B). Differences in root lengths between the mutants and wild-type seedlings grown on 1/2 MS solid media were greatest at 40 ppm MMS (Figure 4C). Leaf number was significantly higher in mutants than in wild-type seedlings exposed to 55 ppm MMS on B5 solid media (Figure 4D) and this effect was accompanied by an increase in overall plant biomass (Figure 4E). We also examined the response of *atsrp2 *and *atsrp3 *mutants to UV light and γ-radiation, but no differential effects (vs. wild-type) could be detected (Figure 5A and 5B). RT-PCR using RNA isolated from whole seedlings (5-d) exposed to γ-radiation showed expression levels of the DNA repair gene *AtRAD51 *were the same in the mutants and wild-type (Figure 5C).

### Induction of DNA repair- and cell cycle-related genes is reduced in *atsrp2 *and *atsrp3 *mutants exposed to MMS

**Figure 6:**
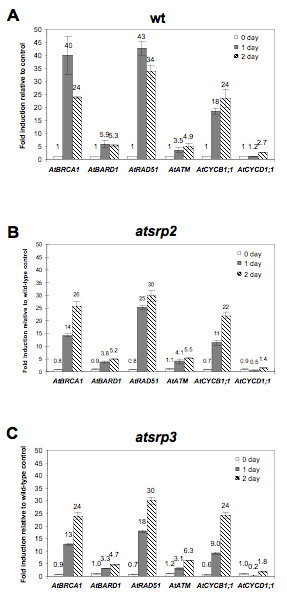
**Expression of DNA damage- and cell cycle-related genes in wild-type and mutants *atsrp2 *and *atsrp3 *following MMS treatment**. Seedlings (5-d) were transplanted to MS liquid media containing 200 ppm MMS and samples collected at the indicated time points. Expression of marker genes in (A) wild-type, (B) *atsrp2 *and (C) *atsrp3 *seedlings was determined by quantitative RT-PCR. Values are means +/- SE (n = 2).

After MMS treatment of seedlings grown on 1/2 MS liquid media, samples were collected at various time points, total RNA was isolated and real-time RT-PCR performed. In wild-type plants, exposure to 200 ppm MMS led to a substantial induction after 1 d of all marker genes examined, with the exception of *AtCYCD1;1*, which was induced after 2 d (Figure 6B and 6C; compare to Figure 6A). Interestingly, for both *atsrp2 *and *atsrp3 *mutants, expression of all marker genes except *AtATM *was lower than that in wild-type plants 1 d after MMS treatment (Figure 6A–C). The cell-cycle related gene *AtCYCD1;1 *was down-regulated 1 day after plant exposure to MMS compared to both the zero time point and to wild-type levels (Fig. 6A–C). Recovery of the expression of all of the marker genes except *AtCYCD1;1 *to wild-type levels occurred by day 2 (Figure 6A–C). The rapid changes in expression of these key genes suggest *AtSRP2 *and *AtSRP3 *function in responses to DNA-damage after exposure to MMS.

## Discussion

### Diversity of *Arabidopsis *serpin reactive centres and differential expression

The reactive centres of the *Arabidopsis *serpins are highly diverse (Figure 1), suggesting a range of target proteinases and therefore distinct biochemical pathways in which the serpins might participate. The only specific target proteinase suggested for a plant serpin to date is the cysteine proteinase Metacaspase 9 (AtMC9) for AtSerpin1 [11]. Differential expression of the serpin genes (Figure 2A and 2B) is corroborated by AtProteome data [14] and provides more evidence for functional diversity. The nuclear localization of AtSRP2 is consistent with predictions [5] based on WoLF PSORT and contrasts with the cytosolic localization of AtSRP3 (Figure 2D). AtSRP2 contains the nuclear localization signal PKKK at the distal end of the RCL (Figure 1); the one-residue difference between this and the corresponding sequence in AtSRP3, PPKK, appears sufficient to retain the latter serpin in the cytosol.

### Responses of *AtSRP2 *and *AtSRP3 *are specific to MMS-induced DNA damage

A test of cross-sensitivity of a collection of MMS-sensitive yeast mutants to hydroxyurea, UV light and γ-radiation found only 41 of 103 mutants showed cross-sensitivity to all three treatments [15]. Deficiency in ATM (ataxia telangiectasia mutated gene) in plants and mammals confers hypersensitivity to γ-radiation and MMS but not to UV light [16,17]. These and other studies show different sources of DNA damage cause distinct types of lesions and forms of cell cycle arrest. This is consistent with the differential responses of our serpin mutants (vs. wild-type) to MMS but not to UV-C or γ-radiation.

### Knockouts of *AtSRP2 *and *AtSRP3 *transiently reduce responses to MMS exposure

Mutants *atsrp2 *and *atsrp3 *exposed to MMS displayed lower levels of induction of *AtBRCA1*, *AtBARD1*, *AtRAD51 *and *AtCYCB1;1*, as well as a downregulation of *AtCYCD1;1*, after 1 d compared to wild-type. Expression levels returned to wild-type levels 2 d after MMS exposure (Figure 6A–C). Thus AtSRP2 and AtSRP3 may be required for relatively early responses to alkylation damage. In humans, *BRCA1 *appears to be involved in all phases of the cell cycle [18] and BRCA1 deficiency causes abnormalities in the S-phase and G2/M checkpoint [19]. RAD51 is involved in homologous DNA repair and interacts with BRCA1 [20]; moreover, transcription of *RAD51 *(and *AtRAD51*) is highest in S-phase [21]. Yeast is most sensitive to MMS during S-phase [22]. Thus down-regulation of *AtBRCA1 *and *AtRAD51 *in the *atsrp2 *and *atsrp3 *mutants may be associated with abnormal S-phase and a defective G2/M checkpoint.

Transcripts of mitotic cyclin *AtCYCB1;1 *are reported to accumulate around the G2/M transition [23], whereas *AtCYCD1;1 *promotes not only transition through G0/G1/S but also S/G2/M [24]. Cyclins (type D) are expressed throughout the cell cycle in proliferating plant tissues [25]. The CYCD1 interaction with cyclin-dependent protein kinase A (CDKA) acts at the G1/S and the G2/M boundaries in *Arabidopsis *[24]. These results suggest *AtSRP2 *and *AtSRP3 *may be involved in the regulation of different cell-cycle checkpoints in response to MMS-induced DNA damage. Interestingly, we detected no difference in *AtATM *expression between mutants and wild-type (Figure 6A–C). The *Arabidopsis atm *mutant is hypersensitive to γ-radiation and MMS but not to UV light [17]. The functions of AtSRP2 and AtSRP3 may be overlapping, as is found for AtATM and AtATR [26], and they may be independent of AtATM in a DNA damage-response pathway; alternatively, both serpins may act downstream of AtATM.

Regulation of the cell cycle includes mechanisms for cell cycle arrest following DNA damage to allow time for repair of the DNA [1]. Failure of these checkpoint responses leads to a build up of harmful mutations. Cell cycle arrest results in reduced rates of growth and a delay in the development of tissues. Lesions such as specific gene knockouts can interfere with the signal transduction required for the detection of DNA damage and responses including cell cycle arrest. We observed greater rates of growth of our *atsrp2 *and *atsrp3 *mutant plants exposed to MMS than that of wild-type plants given the same treatment. Thus we speculate that knockouts of these serpin genes result in de-repression of cell division following MMS treatment, thereby implicating AtSRP2 and AtSRP3 in the normal signal transduction required for the relevant checkpoint responses. It is important to note that Hefner et al. (2006) found that plant cell-cycle responses to ionizing radiation that were observed in meristematic tissues did not occur in strictly somatic tissues [27].

### Serpin-mediated control of proteolysis and its links to DNA damage responses

Sequence analysis strongly suggests AtSRP2 and AtSRP3 are inhibitory serpins [5,6]. AtSerpin1 can inhibit AtMC9, which is Arg/Lys specific [11]. In contrast, we predict AtSRP2 and AtSRP3 inhibit proteinases that cleave at small residues (Figure 1). Since AtSRP2 is localized to the nucleus, its candidate targets are different than those for AtSRP3, which was localized to the cytosol (Figure 2D). Seventeen expressed genes encoding serpins with either P1 Gly or P1 Cys were recently identified from several monocots and eudicots [5]. Thus it is likely that serpins in other plant species are functional homologues of AtSRP2 and AtSRP3.

## Conclusion

The inhibitory serpins encoded in the *Arabidopsis *genome display a variety of reactive centres, suggesting a range of target proteinases, and are differentially expressed, indicating a diversity of functions. Our results strongly suggest a new role for plant serpins, which is likely to involve inhibition of specific endogenous proteinases that target Gly and Cys residues to regulate plant responses to alkylating DNA damage. AtSRP2 and AtSRP3 are found in the cytosol and nucleus, respectively, and thus may perform distinct tasks in the signalling required for these responses. The requirement for AtSRP2 and AtSRP3 in responses to exposure to alkylating genotoxins but not to UV-C or γ-radiation provides support for distinct biochemical pathways associated with these stresses. We suggest our data have potential importance for development of crops with greater resistance to alkylating genotoxins.

## Methods

### Plant materials and treatments with MMS, UV-C and γ-radiation

Seeds of *Arabidopsis thaliana *(Col-0) were sterilized in 4% sodium hypochlorite for 5 min and rinsed five times with sterilized water. Plants were grown in a chamber at 24°C (16 h light/8 h dark). For MMS treatment, B5 medium (Gamborg's B5 medium [28]; Gibco) containing 2% sucrose and 1/2 MS medium (half-strength Murashige & Skoog medium [29]; Sigma) containing 0.5% sucrose were used. Seedlings (5-d) on sterile media (both types) were transplanted into 5 ml of each liquid medium containing zero to 100 ppm MMS (Sigma). Samples were incubated for 15 d on an orbital shaker with constant light. For measurement of root lengths, seedlings (5-d) were transplanted into each solid medium containing MMS and measurements made 15 d later. Seedlings (2 weeks old) were transplanted to media containing MMS for measurements of plant size and leaf number made 4 weeks later. Seedlings (5-d) were exposed to UV-C light (1 and 10 J cm^-1^) in open Petri dishes using a Stratalinker 2400 (Stratagene). For γ-radiation treatment, seedlings (5-d) were irradiated with zero to 200 Gy using a ^60^Co source and then placed into a growth chamber at 24°C for 3 weeks.

### RNA isolation and RT-PCR

Total RNA from *Arabidopsis *tissues was extracted using the RNeasy Plant Mini kit (Qiagen) with 1 U RNase-free DNase (Qiagen). Reverse transcription was performed using 5 μg total RNA as described [30]. cDNAs were used as templates for semi-quantitative RT-PCR and real-time RT-PCR. Primer sequences for all experiments are given in Additional File 1 (Tables one-five). RT-PCR products were cloned and sequences confirmed.

### Isolation of knockout mutants

Seeds from *atsrp2 *and *atsrp3 *T-DNA insertion mutants were obtained from the Salk Institute Genomic Analysis Laboratory. For selection of lines homozygous for the T-DNA insertion, primers a-f and LB were used to amplify left border-flanking and serpin DNA from genomic DNA. For confirmation of knockouts of *AtSRP2 *and *AtSRP3 *expression in *atsrp2 *and *atsrp3 *mutants, semi-quantitative RT-PCR was performed using total RNA from developing siliques.

### Subcellular localization

*AtSRP2 *and *AtSRP3 *full-length cDNAs were amplified by PCR and cloned into a 326-GFP plasmid such that GFP was joined to the N-terminus of the serpins. Fusion constructs expressed under control of the cauliflower mosaic virus 35S promoter were introduced into *Arabidopsis *protoplasts isolated from seedlings (10-d) by polyethylene glycol-mediated transformation. The protoplasts were incubated at 24°C for 24 h. Expression of fusion proteins was observed using a Leica TCS SP2 confocal system.

## Abbreviations used

MMS: methyl methanesulfonate; RCL: reactive centre loop; DSB: double-stranded break; DAPI: 4',6-diamidino-2-phenylindole.

## Authors' contributions

J-WA designed and performed all experimental work and helped to draft the manuscript. BJA and THR gave advice on experimental design and interpretation of results, and drafted the manuscript.

## Supplementary Material

Additional file 1 **Primer sequences**. Primer sequences used for isolation of homozgous T-DNA insertion mutants, RT-PCR for expression analysis and cloning for subcellular localization. Click here for file

## References

[B1] Kimura S, Sakaguchi K (2006). DNA repair in plants. Chem Rev.

[B2] Knight AW, Keenan PO, Goddard NJ, Fielden PR, Walmsley RM (2004). A yeast-based cytotoxicity and genotoxicity assay for environmental monitoring using novel portable instrumentation. J Environ Monit.

[B3] Lundin C, North M, Erixon K, Walters K, Jenssen D, Goldman AS, Helleday T (2005). Methyl methanesulfonate (MMS) produces heat-labile DNA damage but no detectable in vivo DNA double-strand breaks. Nucl Acids Res.

[B4] Rawlings ND, Morton FR, Kok CY, Kong J, Barrett AJ (2008). MEROPS: the peptidase database. Nucl Acids Res.

[B5] Roberts TH, Hejgaard J (2008). Serpins in plants and green algae. Funct Integr Genomics.

[B6] Hejgaard J, Roberts TH, Silverman GA, Lomas DA (2007). Plant serpins. Molecular and Cellular Aspects of the Serpinopathies and Disorders in Serpin Activity.

[B7] Huntington JA, Read RJ, Carrell RW (2000). Structure of a serpin-protease complex shows inhibition by deformation. Nature.

[B8] Ostergaard H, Rasmussen SK, Roberts TH, Hejgaard J (2000). Inhibitory serpins from wheat grain with reactive centers resembling glutamine-rich repeats of prolamin storage proteins – Cloning and characterization of five major molecular forms. J Biol Chem.

[B9] Hejgaard J (2001). Inhibitory serpins from rye grain with glutamine as P-1 and P-2 residues in the reactive center. FEBS Lett.

[B10] Hejgaard J, Laing WA, Marttila S, Gleave AP, Roberts TH (2005). Serpins in fruit and vegetative tissues of apple (*Malus domestica*): expression of four serpins with distinct reactive centres and characterisation of a major inhibitory seed form, MdZ1b. Funct Plant Biol.

[B11] Vercammen D, Belenghi B, Cotte B van de, Beunens T, Gavigan JA, De Rycke R, Brackenier A, Inze D, Harris JL, Van Breusegem F (2006). Serpin1 of *Arabidopsis thaliana *is a suicide inhibitor for Metacaspase 9. J Mol Biol.

[B12] Yoo BC, Aoki K, Xiang Y, Campbell LR, Hull RJ, Xoconostle-Cazares B, Monzer J, Lee JY, Ullman DE, Lucas WJ (2000). Characterization of *Cucurbita maxima *phloem serpin-1 (CmPS-1) – A developmentally regulated elastase inhibitor. J Biol Chem.

[B13] Schechter I, Berger A (1967). On the size of the active site in proteases. I. Papain. Biochem Biophys Res Commun.

[B14] Baerenfaller K, Grossmann J, Grobei MA, Hull R, Hirsch-Hoffmann M, Yalovsky S, Zimmermann P, Grossniklaus U, Gruissem W, Baginsky S (2008). Genome-scale proteomics reveals *Arabidopsis thaliana *gene models and proteome dynamics. Science.

[B15] Chang M, Bellaoui M, Boone C, Brown GW (2002). A genome-wide screen for methyl methanesulfonate-sensitive mutants reveals genes required for S phase progression in the presence of DNA damage. Proc Natl Acad Sci USA.

[B16] Gasch AP, Huang M, Metzner S, Botstein D, Elledge SJ, Brown PO (2001). Genomic expression responses to DNA-damaging agents and the regulatory role of the yeast ATR homolog Mec1p. Mol Biol Cell.

[B17] Garcia V, Bruchet H, Camescasse D, Granier F, Bouchez D, Tissier A (2003). AtATM is essential for meiosis and the somatic response to DNA damage in plants. Plant Cell.

[B18] Deng CX (2006). BRCA1: cell cycle checkpoint, genetic instability, DNA damage response and cancer evolution. Nucl Acids Res.

[B19] Xu X, Weaver Z, Linke SP, Li C, Gotay J, Wang XW, Harris CC, Ried T, Deng CX (1999). Centrosome amplification and a defective G2-M cell cycle checkpoint induce genetic instability in BRCA1 exon 11 isoform-deficient cells. Mol Cell.

[B20] Pellegrini L, Yu DS, Lo T, Anand S, Lee M, Blundell TL, Venkitaraman AR (2002). Insights into DNA recombination from the structure of a RAD51-BRCA2 complex. Nature.

[B21] Doutriaux MP, Couteau F, Bergounioux C, White C (1998). Isolation and characterisation of the RAD51 and DMC1 homologs from *Arabidopsis thaliana*. Mol Gen Genet.

[B22] Tercero JA, Diffley JF (2001). Regulation of DNA replication fork progression through damaged DNA by the Mec1/Rad53 checkpoint. Nature.

[B23] Doerner P, Jorgensen JE, You R, Steppuhn J, Lamb C (1996). Control of root growth and development by cyclin expression. Nature.

[B24] Koroleva OA, Tomlinson M, Parinyapong P, Sakvarelidze L, Leader D, Shaw P, Doonan JH (2004). CycD1, a putative G1 cyclin from *Antirrhinum majus*, accelerates the cell cycle in cultured tobacco BY-2 cells by enhancing both G1/S entry and progression through S and G2 phases. Plant Cell.

[B25] Sorrell DA, Combettes B, Chaubet-Gigot N, Gigot C, Murray JA (1999). Distinct cyclin D genes show mitotic accumulation or constant levels of transcripts in tobacco bright yellow-2 cells. Plant Physiol.

[B26] Culligan KM, Robertson CE, Foreman J, Doerner P, Britt AB (2006). ATR and ATM play both distinct and additive roles in response to ionizing radiation. Plant J.

[B27] Hefner E, Huefner N, Britt AB (2006). Tissue-specific regulation of cell-cycle responses to DNA damage in *Arabidopsis *seedlings. DNA Repair.

[B28] Gamborg OL, Miller RA, Ojima K (1968). Nutrient requirements of suspension cultures of soybean root cells. Exp Cell Res.

[B29] Murashige T, Skoog F (1962). A revised medium for rapid growth and bioassays with tobacco tissue cultures. Physiol Plant.

[B30] Kim M, Ahn JW, Jin UH, Choi D, Paek KH, Pai HS (2003). Activation of the programmed cell death pathway by inhibition of proteasome function in plants. J Biol Chem.

