# Supplementary material for: Serpin genes AtSRP2 and AtSRP3 are required for normal growth sensitivity to a DNA alkylating agent in Arabidopsis
Source: BMC Plant Biol. 2009 May 11;9:52. doi: 10.1186/1471-2229-9-52 (PMC2689219; doi:10.1186/1471-2229-9-52)
Supplement: Additional file 1 — Primer sequences. Primer sequences used for isolation of homozgous T-DNA insertion mutants, RT-PCR for expression analysis and cloning for subcellular localization. [file 1471-2229-9-52-S1.doc]

**Table 1**

Primers sequences for Figure 2A, B and Figure 3E

| At1g62170 | 5′-ATGGAGCCAAAAGAGAAGAAA-3′ |
| --- | --- |
|  | 5′-Ctaagcagaaggatcgaa-3′ |
| *AtSRP2* | 5′-ATGGATTCAAAAAGAAAGAAC-3′ |
|  | 5′-ttagcccggtccaacgca-3′ |
| *AtSRP3* | 5′-ATGGATGTAAGAGAAGCT-3′ |
|  | 5′-ttaatagtcatctgagtc-3′ |
| At3g45220 | 5′-ATGGAGTTGGGAAAATCAATG-3′ |
|  | 5′-atccaattaatgtattga-3′ |
| At2g26390 | 5′-ATGGAGTTAGGAAAATCAATT-3′ |
|  | 5′-gttcaatcaatgtttaga-3′ |
| *Atserpin1* | 5′-ATGGACGTGCGTGAATCAATC-3′ |
|  | 5′-ttaatgcaacggatcaac-3′ |
| *Actin2* | 5′-TCGGTGGTTCCATTCTTGCT-3′ |
|  | 5′-GCTTTTTAAGCCTTTGATCTTGAGAG-3′ |

**Table 2**

Primers sequences for Figure 2C

| *AtSRP2* | 5′-TACCGCGTTGATGTTGGTG-3′ |
| --- | --- |
|  | 5′-TACCAAACCCTTATCCGGT-3′ |
| *AtSRP3* | 5′-GATTTCCGATCAGAGGCTG-3′ |
|  | 5′-gacaaggtagaagtcattatc-3′ |

**Table 3**

Primer sequences for Figure 2D

| *AtSRP3* | 5′-ATCCCCGGGCAATGGATGTAAGAGAAGCT-3′ |
| --- | --- |
|  | 5′-Actgtcgacttaatagtcatctgagtc-3′ |
| *AtSRP2* | 5′-ATCCCCGGGCAATGGATTCAAAAAGAAAGAAC-3′ |
|  | 5′-Actgtcgacttagcccggtccaacgca-3′ |

**Table 4**

| primer d | 5′-ATGGATGTAAGAGAAGCT-3′ |
| --- | --- |
| primer e | 5′-tatagagatgaacttgaga-3′ |
| primer f | 5′-ttaatagtcatctgagtc-3′ |
| primer a | 5′-ATGGATTCAAAAAGAAAGAAC-3′ |
| primer b | 5′-taccgcgttgatgttggtg-3′ |
| primer c | 5′-ttagcccggtccaacgca-3′ |
| LB | 5′-GCGTGGACCGCTTGCTGCAAC-3′ |

Primer sequences for Figure 3A-D

**Table 5**

Primer sequences for Figure 5C and Figure 6

| *AtATM* | 5′-gcaaagacagaaggaaac-3′ |
| --- | --- |
|  | 5′-CAGCCGAGTATTTTTCAAC-3′ |
| *AtCYCB1;1* | 5′-TCAGTCCATCAATGGTAG -3′ |
|  | 5′-TCAAAGCCACAGCGAAGC -3′ |
| *AtBARD1* | 5′-GAACTGGCAAGGGGGATG-3′ |
|  | 5′-TCGTTCGGGAATTTGACAG-3′ |
| *AtBRCA1* | 5′-TCTTGGAAGACAAAGAGC-3′ |
|  | 5′-AAGAAACAGGCCGCCTAC-3′ |
| *AtRAD51* | 5′-tcgatagtgctaccgctc-3′ |
|  | 5′-TCAACGCCAACCTTGTTG-3′ |
| *AtCYCD1;1* | 5′-CATTAGAGTCCTCATCAC-3′ |
|  | 5′-TCCGTCACGTGAAGTCAC-3′ |
| *Actin2* | 5′-TCGGTGGTTCCATTCTTGCT-3′ |
|  | 5′-GCTTTTTAAGCCTTTGATCTTGAGAG-3′ |
